# Conduct Symptoms and Emotion Recognition in Adolescent Boys with Externalization Problems

**DOI:** 10.1155/2013/826108

**Published:** 2013-11-04

**Authors:** Nikoletta Aspan, Peter Vida, Julia Gadoros, Jozsef Halasz

**Affiliations:** ^1^Vadaskert Child Psychiatry Hospital, 5 Lipotmezei Street, Budapest 1021, Hungary; ^2^School of Ph.D. Studies, Semmelweis University, 26 Ulloi Street, Budapest 1085, Hungary; ^3^Alba Regia University Centre, Obuda University, 45 Budai Street, Szekesfehervar 8000, Hungary

## Abstract

*Background*. In adults with antisocial personality disorder, marked alterations in the recognition of facial affect were described. Less consistent data are available on the emotion recognition in adolescents with externalization problems. The aim of the present study was to assess the relation between the recognition of emotions and conduct symptoms in adolescent boys with externalization problems. *Methods*. Adolescent boys with externalization problems referred to Vadaskert Child Psychiatry Hospital participated in the study after informed consent (*N* = 114, 11–17 years, mean = 13.4). The conduct problems scale of the strengths and difficulties questionnaire (parent and self-report) was used. The performance in a facial emotion recognition test was assessed. *Results*. Conduct problems score (parent and self-report) was inversely correlated with the overall emotion recognition. In the self-report, conduct problems score was inversely correlated with the recognition of anger, fear, and sadness. Adolescents with high conduct problems scores were significantly worse in the recognition of fear, sadness, and overall recognition than adolescents with low conduct scores, irrespective of age and IQ. *Conclusions.* Our results suggest that impaired emotion recognition is dimensionally related to conduct problems and might have importance in the development of antisocial behavior.

## 1. Introduction

Individuals with antisocial personality disorder express failure to conform to social norms, deceitfulness, impulsivity, irritability and/or aggressiveness, reckless disregard for safety, consistent irresponsibility, lack of remorse, and “a pervasive disregard for and violation of the right of others” [1]. Both genetic and environmental factors were extensively studied in the formation of antisocial behavior [2, 3]. Externalization symptoms are common in children and adolescents and indicate vulnerability for later antisocial behavior [4]. For example, the cumulative prevalence of conduct disorder in boys by the age of 16 years is about ten percent, often interrelated with other externalization problems [5]. The presence of conduct disorder, psychopathic traits, and callous/unemotional traits has major importance in the development of antisocial personality disorder [4, 6].

Impairment in the recognition of facial effect might create, enlarge, and maintain behavioral problems during development. A meta-analysis of Marsh and Blair showed a robust link between adult antisocial behavior and specific deficits in recognizing fearful expressions [7]. The meta-analysis predominantly analyzed studies including antisocial males, as the majority of antisocial literature per se deals with gender bias. Additionally, impaired recognition of sadness was also indicated, but smaller effect size was present. Consistent alteration in the recognition of anger, disgust, happiness, and surprise was not found [7].

Compared to the results described in antisocial individuals, literature is less conclusive in the recognition of emotions in children and adolescents with externalization problems. Early studies highlighted the importance of psychopathic traits in boys, and impairment in the recognition of facially expressed fear and sadness was described [8, 9]. A study on adolescent males with conduct disorder could confirm the alteration in the recognition of fear and sadness comparing individuals with high and low psychopathic traits and indicated subtle differences between adolescent boys with early-onset and adolescent-onset conduct disorder [10]. Dadds and colleagues even described that impaired fear recognition in individuals with high level of psychopathic traits was accompanied by a reduction of gaze directed toward periorbital regions [11]. Other groups could not confirm the alteration in the recognition of fear and sadness in boys with psychopathic tendencies [12, 13]. In girls, externalizing problems might be differently associated with emotion recognition. In girls with conduct disorder, higher psychopathic traits were associated with impaired recognition of sadness [14] and even increased recognition of fear [15]. The presence of conduct disorder in girls was accompanied by impaired recognition of anger and disgust [14], sadness [15], and no major effect [16]. In a mixed gender sample of children and adolescents with depression, the presence of conduct disorder did not modify emotion recognition [17].

It seems that gender, the presence of conduct disorder, comorbidities, and psychopathic trait have importance in the alterations of facial affect recognition in children and adolescents. Interestingly, dimensional approaches were rarely applied in the above papers to assess the relationship between externalizing problems and emotion recognition. For example, Schwenck et al. made a correlation between the number of conduct symptoms and the recognition of emotions [15]. In the above paper, the measures of the widely used Child Behavior Checklist were also assessed, but correlation data with the recognition of emotions were not published. In a recent paper, an inverse correlation between conduct problems and emotion recognition (overall emotion recognition and fear) in nonclinical adolescent boys (but not in girls) was described, suggesting a dimensional link between behavioral and neuropsychological measures in boys [18].

The aim of the present paper was to assess the relationship between conduct problems and emotion recognition in boys with externalization problems. We hypothesized an inverse correlation of the recognition of sad and fearful expressions with the Strengths and Difficulties Questionnaire (SDQ) conduct problems score in clinically referred adolescent boys with externalization problems.

## 2. Methods

### 2.1. Participants

During the study, 123 adolescent boys (aged between 11–17) with externalization problems referred to the Vadaskert Child Psychiatry Hospital were involved voluntarily after informed consent. The Vadaskert Child Psychiatry Hospital accepts children and adolescents with different behavioral problems from the whole country (Hungary) and provides both in- and outpatient services. The study was conducted in the Vadaskert Child Psychiatry Hospital, with the permission of the joint Ethical Committee of the Saint John and the Saint James Hospitals. According to the Hungarian guidelines, written informed consent from both the adolescent and at least one parent was obligatory to participate in the study. 

The final sample consisted of 114 boys (age: 13.40 ± 0.16 years, mean ± SEM). The data of nine adolescents (from the original 123) were not used because of the exclusion criteria. The exclusion criteria were (i) Raven IQ below 80 (five adolescents had lower Raven IQ), (ii) current or past psychotic experience (one adolescent), and (iii) pervasive developmental disorder (three adolescents), and thus their data were not used in the study. Past psychotropic medication was not among the exclusion criteria. Documented previous (any time) psychotropic medication was present in 44 cases (38.6%). The majority of previous medication was methylphenidate (34 cases, 29.8%; in 1 case in combination with hydroxyzine and in 1 case with haloperidol), fluvoxamine (2 cases), imipramine (2 cases), risperidone (2 cases), chlorprothixene (1 case), clomipramine (1 case), hydroxyzine (1 case), and sertraline (1 case). Adolescents were medication-free at least for 72 hours before the emotion recognition task. Previous psychotherapy was present in 56 cases (49.1%). Among the participants, 88 (77.2%) had clinical disruptive behavioral disorder. In the case of 63 adolescents (53.5%), parents were divorced or separated. Living with adopting parents and/or foster care was present at 16 adolescents (14.0%). The wider living environment of the adolescents was smaller town (47.4%, less than 10000 citizens), county capital (38.6%), and middle town (14%). 

### 2.2. Procedure

After informed consent, adolescents participated in a test of emotion recognition and their performances in the Raven's Progressive Matrices were also assessed. Behavioral data were obtained both from parents and the adolescents by the means of the SDQ. Additional to the regular clinical interview and diagnosis formulation, the procedure lasted about 60–70 minutes for the participants.

### 2.3. Instruments

#### 2.3.1. The SDQ

The SDQ is widely used as a general questionnaire on the behavioral problems of children and adolescents. The original, parent/teacher version of the SDQ was published in 1997 by Goodman [19], and later a self-reported version was also created [20]. The SDQ is used both in clinical and population research. It can be used for early screening, testing subclinical symptoms, and estimating functional impairment [21]. The Hungarian translation and the validation of the parent version in general sample were performed in 2008 [22], and further confirmation in adolescents and the validation of the self-report version were published in 2011 [23]. The clinical validation of the SDQ in Hungarian population was published recently [24]. Both the parent and self-report versions of the SDQ contain 25 items, and the questionnaire consists five scales (emotional symptoms, conduct problems, hyperactivity, peer problems, and prosocial scale). For the present research, only the data from conduct problems scale was used. In the questionnaire, a Likert scale is used (0—not true, 1—somewhat true, 2—certainly true). The items of the conduct problems scale result a maximum of 10 points (items: often has temper tantrums or hot tempers; generally obedient, usually does what adults request (inverted item); often fights with other children and bullies them; often lies or cheats; steals from home, school, or elsewhere).

#### 2.3.2. The Recognition of Emotions (Facial Expressions of Emotion-Stimuli and Tests (FEEST))

Based on the six universal basic emotions described by Ekman and colleagues [25], the computerized and extended version of the original 60 faces test—FEEST—was used [26]. The “Emotion” test was used in the study. During the procedure, adolescents choose a label for the emotional content (anger, disgust, fear, happiness, sadness, and surprise) of the faces visible on the screen. The maximum number of correct responses for each emotions was 10, altogether 60.

#### 2.3.3. Raven's Test of Progressive Matrices

Standard Progressive Matrices were used [27]. The 60 progressive steps were standardized according to the Hungarian gender and age, and a general IQ measure was outlined. The Raven performance scores were between 80 and 158 (109.1 ± 1.0, mean ± SEM).

### 2.4. Statistical Analysis

The analysis was performed with the SPSS 20.0 software package. Spearmans correlation was used to assess the relationship between conduct problems scores and variables in emotion recognition. General linear model was used to assess the effect of conduct scores on the recognition of emotions (the effects of sociodemographic variables, age, and Raven IQ were also tested); the six basic emotions and overall measures were also analyzed. The level of significance was set at *P* < 0.05. 

## 3. Results

### 3.1. SDQ Conduct Problems Scale

Among the 114 boys referred for externalization problems, 95 (83.3%) reached the level of clinical problems at the conduct problems scale (above two points, according to the British and the Hungarian standards) in the parent version of the SDQ (5.0 ± 0.2, mean ± SEM), while similar measure in the self-report version of the SDQ was found in 63 adolescents (55.3%, above three points; 3.9 ± 0.2, mean ± SEM).

### 3.2. Emotion Recognition and Conduct Problems Scale

The SDQ conduct problems score was inversely correlated with the overall emotion recognition (parent report: *R* = −0.23; *P* < 0.02; self-report: *R* = − 0.21; *P* < 0.02). The SDQ conduct problems score (self-report) was inversely correlated with the recognition of anger (*R* = − 0.19; *P* < 0.05), fear (*R* = − 0.19; *P* < 0.05), and sadness (*R* = − 0.30; *P* < 0.001).

**Figure 1:**
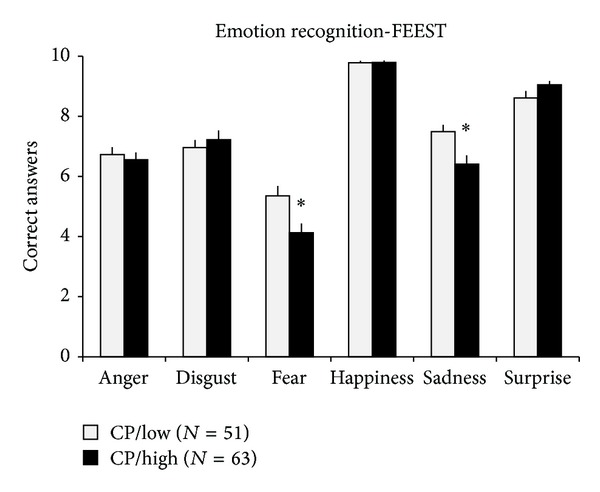
Results from the emotion recognition task (facial expressions of emotion-stimuli and tests (FEEST)) are presented. Data are expressed as mean ± SEM. CP/low: group of adolescent boys where low scores of conduct problems in the self-report version of the SDQ were present; CP/high: group of adolescent boys where high scores of conduct problems in the self-report version of the SDQ were present; ∗: statistically significant difference (*P* < 0.05) from CP/low group.

Two groups were created according to the SDQ conduct problems self-report scores (below or above the clinical standards, low and high scores on the SDQ conduct problems scale, CP/low and CP/high groups, resp.). The adolescents in the CP/high group showed a significantly worse performance compared to CP/low group in the overall emotion recognition (*F*_(1,112)_ = 4.53; *P* < 0.04; CP/low: 44.9 ± 0.6, CP/high: 43.2 ± 0.5, mean  ±  SEM). Similarly, the CP/high group showed a significantly worse performance compared to CP/low group in the recognition of fear (*F*_(1,112)_ = 7.48; *P* < 0.01) and sadness (*F*_(1,112)_ = 7.97; *P* < 0.01), while the recognition of other emotions was not different between groups (Figure 1). The difference in the recognition of fear and sadness could not be attributed to the age, Raven IQ, or sociodemographic variables of the participants.

## 4. Discussion

### 4.1. Main Findings

The main results of the present study were that (i) overall emotion recognition was inversely correlated with conduct problems (according to parent and self-report SDQ conduct problems scores) and (ii) adolescents with high conduct problems were significantly worse in the recognition of emotions on faces expressing fear and sadness, irrespective of age, IQ, and sociodemographic measures.

### 4.2. Emotion Recognition in the Antisocial Pathway

Data on the emotion recognition of adult antisocial males consistently represent a large bias in the recognition of fear as was excellently described in a meta-analysis involving more than 20 studies [7]. Data are less pronounced in the case of adolescent males with conduct disorder. The majority of adolescent data on emotion recognition did not study conduct disorder or conduct symptoms directly but investigated the presence of psychopathic traits or callous/unemotional traits. High psychopathic traits were associated with decreased recognition of fear [8, 9], but data not supporting this assumption were also published [13]. In the study of Fairchild et al., fear recognition was decreased in adolescent boys with conduct disorder [10]. Our study supports these data in wider context, as conduct problems were inversely correlated with the recognition of fear in adolescent boys with externalization problems. In an earlier study, conduct problems were inversely related with the recognition of fear in nonclinical adolescent boys (but not in girls) [18]. It seems that this correlation also exists in clinically referred boys. 

The recognition of fear among basic emotions seems the most difficult even for healthy individuals, and this natural bias seems consecutively enlarged in individuals with antisocial personality disorder [7, 28]. According to literature data, the impairment in the recognition of fear suggests dysfunctions in specified neural substrates (amygdala) involved in processing fearful facial effect [7, 29]. Both basic [30–32] and clinical studies [33–36] outline the importance of amygdala and amygdalo-prefrontal interplay in the occurrence of aggressive behavior. In fMRI studies, in adolescent boys with conduct disorder and high callous/unemotional traits decreased amygdalar activation [34, 35] and decreased amygdalo-prefrontal coupling were present on images of faces expressing fear [34]. It seems that the amygdalo-prefrontal dysfunction which is considered crucial in the formation of antisocial behavior is highly important in the bias of emotion recognition, especially in the recognition of fear.

### 4.3. Intervention Strategies

At present, according to the NICE guideline, no “A” evidence (neither psychotherapeutic nor pharmacotherapeutic) treatment exists in antisocial personality disorder [37]. Data suggest that robust neuropsychological alterations, difficulties in the recognition of facial affect, discrepancies in emotional decision making, and disturbances in both cognitive and affective components of empathy exist in individuals with antisocial personality disorder [6]. Efficient and powerful early interventions targeting these specific neuropsychological alterations might be beneficial in the prevention of the full development of antisocial behavior. Thus, research questions addressing emotion recognition during antisocial pathway might have therapeutic importance during antisocial development. Decreased fear recognition in boys with high psychopathic traits and externalization problems was associated with a decreased gaze directed towards the periorbital regions measured by eye-tracking system [11]. In the above study, instructing children for screening the periorbital region during the task significantly improved the performance on the recognition of fear [11]. First data on possible interventional strategies have already been published [38]. Impairment in the emotion recognition might result in serious bias in the interpretation of social signal, alter cognitive and affective components of empathy, and result in serious deficits in social behavior [38]. Our study supported a dimensional link between behavioral and neuropsychological measures in adolescents with externalization problems and indirectly supports the necessity of intervention studies on emotion recognition in selected individuals. 

### 4.4. Limitations of the Study

The limitations of the study are that (i) only static images were used in the facial emotion recognition process and (ii) no detailed diagnostic interview on clinical conditions and comorbidities was performed. Dynamic and morph-features were also included in certain studies [12, 15], but the majority of literature used static images in the recognition of emotions [8–10]. In the future, parallel application of static and dynamic inputs is planned. The other limitation of the study is the lack of comorbidities within the analysis. The adolescents of the present study were recruited from the regular clinical intervention, and an experienced child psychiatrist and a clinical psychologist confirmed the leading clinical diagnoses of the participants. However, these diagnoses were not used in the present paper, but the widely used SDQ measures were used to describe behavioral profile related to conduct problems. The usage of SDQ in general screening and population studies seems a fruitful strategy for later studies [21].

## 5. Conclusions

Our results indicate a significant interaction of conduct problems and the recognition of emotions in adolescent boys, which supports and extends earlier literature data. “Fear blindness” is a robust phenomenon along antisocial development [7, 11]. Altered emotion recognition seems to be crucial during antisocial development, and the mutual reinforcement between the behavioral and neuropsychological functions might establish important intervention strategies in the future targeting “fear blindness” [38].
